# Prevalence of gestational diabetes mellitus based on various screening strategies in western Kenya: a prospective comparison of point of care diagnostic methods

**DOI:** 10.1186/s12884-017-1415-4

**Published:** 2017-07-14

**Authors:** Sonak D. Pastakia, Benson Njuguna, Beryl Ajwang’ Onyango, Sierra Washington, Astrid Christoffersen-Deb, Wycliffe K Kosgei, Ponnusamy Saravanan

**Affiliations:** 1 Department of Pharmacy Practice, Purdue Kenya Partnership, PO Box 5760, Eldoret, 30100 Kenya; 2 Department of Pharmacy, Moi Teaching and Referral Hospital, PO Box 3, Eldoret, 30100 Kenya; 3 Department of Reproductive Medicine, University of California, San Diego, CA 92103 USA; 4 Department of Obstetrics & Gyneacology, University of Toronto, Toronto, ON M5G 1E2 Canada; 5 Division of Reproductive Health, Moi Teaching and Referral Hospital, PO Box 3, Eldoret, 30100 Kenya; 6 Department of Diabetes, Endocrinology & Metabolism, Warwick Medical School, University of Warwick, Coventry, CV4 7AL UK; 7 Diabetes and Endocrine Centre, George Eliot Hospital, Nuneaton, CV107DJ UK

**Keywords:** Gestational diabetes mellitus, Screening, Prevalence, Diagnosis, Low middle income

## Abstract

**Background:**

Early diagnosis of gestational diabetes mellitus (GDM) is crucial to prevent short term delivery risks and long term effects such as cardiovascular and metabolic diseases in the mother and infant. Diagnosing GDM in Sub-Saharan Africa (SSA) however, remains sub-optimal due to associated logistical and cost barriers for resource-constrained populations. A cost-effective strategy to screen for GDM in such settings are therefore urgently required. We conducted this study to determine the prevalence of gestational diabetes mellitus (GDM) and assess utility of various GDM point of care (POC) screening strategies in a resource-constrained setting.

**Methods:**

Eligible women aged ≥18 years, and between 24 and 32 weeks of a singleton pregnancy, prospectively underwent testing over two days. On day 1, a POC 1-h 50 g glucose challenge test (GCT) and a POC glycated hemoglobin (HbA1c) was assessed. On day 2, fasting blood glucose, 1-h and 2-h 75 g oral glucose tolerance test (OGTT) were determined using both venous and POC tests, along with a venous HbA1c. The International Association of Diabetes in Pregnancy Study Group (IADPSG) criteria was used to diagnose GDM. GDM prevalence was reported with 95% confidence interval (CI). Specificity, sensitivity, positive predictive value, and negative predictive value of the various POC testing strategies were determined using IADPSG testing as the standard reference.

**Results:**

Six hundred-sixteen eligible women completed testing procedures. GDM was diagnosed in 18 women, a prevalence of 2.9% (95% CI, 1.57% - 4.23%). Compared to IADPSG testing, POC IADPSG had a sensitivity and specificity of 55.6% and 90.6% respectively while that of POC 1-h 50 g GCT (using a diagnostic cut-off of ≥7.2 mmol/L [129.6 mg/dL]) was 55.6% and 63.9%. All other POC tests assessed showed poor sensitivity.

**Conclusions:**

POC screening strategies though feasible, showed poor sensitivity for GDM detection in our resource-constrained population of low GDM prevalence. Studies to identify sensitive and specific POC GDM screening strategies using adverse pregnancy outcomes as end points are required.

**Trials registration:**

Clinical trials.gov: NCT02978807, Registered 29 November 2016.

## Background

Gestational diabetes mellitus (GDM) is associated with adverse maternal and fetal outcomes which include macrosomia and subsequent delivery risks such as birth trauma and increased need for caesarian delivery in the short term [1, 2]. Long term, mothers with GDM and their offspring are at an elevated risk for obesity, type 2 diabetes mellitus (T2DM) and other cardiovascular diseases [3–6]. Prompt diagnosis of GDM is the first step towards effective management and prevention of adverse outcomes. GDM case detection in Sub-Saharan Africa (SSA) however, remains sub-standard [7, 8].

The World Health Organization (WHO) adopted criteria developed by the International Association of Diabetes and Pregnancy Study Group (IADPSG) in 2013 for the diagnosis of GDM [9, 10]. This approach however, has certain limitations that limit its applicability in regular diabetes screening in SSA. Firstly, it requires women to come in fasting for an antenatal clinic (ANC) visit between weeks 24–28, posing logistical obstacles such as long distance to clinic and associated transport costs [11, 12]. Furthermore, women who aren’t informed during an earlier visit or forget to come in fasting, require a subsequent visit. Secondly, quality lab infrastructure and reagents for venous glucose testing are not readily available in most SSA health facilities and where available, require operation by skilled and trained personnel who may be unavailable [13–16]. Thirdly, multiple samples required for testing are cumbersome to obtain, both for the woman and healthcare personnel [15]. Finally, associated costs for IADPSG testing are high and may be unaffordable to a majority of low-income patients [14, 17].

Practical and scalable GDM screening and diagnostic tools are urgently required in SSA [18]. Ideal tools to overcome the above limitations should be: accurate in the non-fasting state, avoid need for multiple venous samples, require minimal training to operate, and be cost-effective [15]. Point of care (POC) testing devices possess these characteristics and therefore may be practical tools for GDM screening in a rural/semi-urban SSA setting with limited resources and patient follow-up.

Our study evaluated the accuracy of various POC testing modalities for the diagnosis of GDM compared to the recommended IADPSG testing. We also determined the prevalence of GDM in a previously unstudied rural/semi-urban setting of Western Kenya.

## Methods

Our prospective study enrolled pregnant women from any of three ANC clinics namely: the Moi Teaching and Referral Hospital (MTRH), the Uasin Gishu District Hospital (UGDH), and Mediheal hospital. MTRH is one of only two national referral hospitals in Kenya and serves a varied demography of patients from both rural and urban Western Kenya. UGDH is a small public hospital which primarily serves patients from Eldoret town (semi-urban) and its environs (rural). Mediheal is a private institution primarily serving patients of higher income status from urban Eldoret compared to the other two facilities.

All pregnant women at 24–32 weeks of gestation with singleton pregnancies were eligible for inclusion. We extended the gestational age beyond the recommended 24–28 weeks to provide flexibility for women who did not precisely recall the date of their last normal menstrual period. Gestational age was confirmed using Naegele’s rule and compared to the pregnant woman’s fundal height. Any observed discrepancy of ≥3 weeks was resolved with evaluation by ultrasound. Women were excluded if they had a pre-existing diagnosis of diabetes, were less than 18 years old, were on medications that affect glucose control, or were unable or unwillling to provide informed consent.

Full approval from the Moi University and MTRH institutional research ethics committee (approval number – IREC/2012/66) and the Indiana University-Purdue University institutional review board were acquired prior to study initiation. Written informed consent was obtained from all participants. Baseline demographic and clinical information was obtained for all women using a structured questionnaire administered on enrollment.

Testing procedures were completed over 2 days. Day 1 tests were conducted on the day of enrollment in a non-fasting state. A capillary blood sample was obtained to perform a POC random blood glucose (RBG) test (Optium H®, Abbott Diabetes Care) and glycated hemoglobin (HbA1c) (A1C Now®, PTS Diagnostics). An unexpected stockout necessitated the switch to the desktop DCA Vantage (Siemens®) for HbA1c for 76% of the women. A 50 g glucose load was then administered as a solution from a commercially available glucose product and capillary blood samples were collected at 1-h for POC measurement.

All participants were then expected to return the next day (or within 1 week for those unable to follow-up immediately) after having fasted overnight for ≥8 h. If a patient had a POC 1-h glucose post-50 g load ≥7.78 mmol/L (140 mg/dL) however, they were notified that their result met the threshold for a positive screening and were informed of their need to confirm their diagnosis on their own if they were unable to come back for day 2 testing as part of the study.

**Fig. 1:**
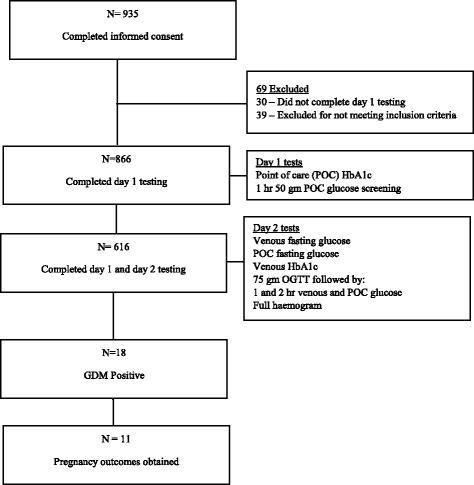
Study schema and patient flow

Day 2 testing procedures included recommended IADPSG testing alongside the analogous POC tests (Fig. 1). Capillary and venous blood samples for measurement of POC and venous fasting blood glucose (FBG) (COBAS® analyzer, Roche Diagnostics), venous HbA1c and a complete blood count with differential was performed. A 75 g glucose load was then administered with capillary and venous blood samples collected at 1-h and 2-h for measurement of POC and venous glucose levels.

All study participants received standard antenatal care offered at the ANC clinics. Pregnant women who were diagnosed with GDM using IADPSG criteria were referred to a home-based glucose monitoring program for care of GDM [19]. GDM positive mothers were also called at least 6 weeks after they had delivered to return to clinic for a fasting blood glucose test to assess whether they had progressed to diabetes, and provide information on their delivery.

For fasting, 1-h and 2-h glucose testing following a 75 g oral glucose tolerance test (OGTT), we utilised glucose cut-offs consistent with IADPSG criteria to define GDM, [20] that is, venous fasting blood glucose (FBG) >5.1 mmol/L (92 mg/dL), or 1-h glucose >10 mmol/L (180.0 mg/dL), or 2-h glucose >8.5 mmol/L (153.0 mg/dL).

We also defined HbA1c values ≥6.5% and RBG ≥ 11.1 mmol/L (199.8 mg/dL) as meeting criteria for GDM diagnosis based on their use as cutpoints for overt diabetes, however, only patients who met IADPSG GDM diagnostic criteria for the OGTT were informed of having a GDM diagnosis and were managed as having GDM [20, 21].

The primary outcomes assessed were the prevalence of GDM determined by IADPSG testing and the sensitivity, specificity, negative predictive value (NPV) and positive predictive value (PPV) of different potential screening strategies when compared to the gold standard 75 g OGTT venous testing recommended by IADPSG. Secondary outcomes includedthe association between POC and corresponding venous testing results, assessed by calculating the mean absolute relative difference (MARD), correlation coefficient, and constructing Bland-Altman graphs for each comparable glucose value. In addition, obstetric outcomes were assessed by contacting all women diagnosed with GDM via phone and completing a standard data collection form which specifically assessed the presence of common GDM related complications. All outcomes were assessed by an independent Obstetrics and Gynaecology specialist to assess the potential association with GDM. Every woman was specifically assessed for fetopelvic disproportion, perineal trauma, preeclampsia, shoulder dystocia, birth injury for baby, congenital malformation, neonatal hypoglycemia, and admission to the neonatal intensive care unit, polycythemia, or hyperbilirubinemia.

The sensitivity, specificity, NPV and PPV of each screening strategy was determined using 2 by 2 matrices with the IADPSG criteria as the gold standard reference. Based on data from prior trials, we structured our sample size calculation to identify a screening strategy that maintained a specificity of 80% and sensitivity of 70% for the three screening strategies we employed. Prior estimates of GDM in SSA are widely distributed with prevalence estimates as low as 0% in some rural regions and as high as 13.9% among high-risk women in urban areas. [8] Considering this wide variability, we conservatively estimated that the prevalence in our semi-urban cohort would be 7%. Based on this assumption, and anticipating a 30% loss-to-follow up, we needed to screen 667 pregnant women to identify 36 cases with GDM between 24 and 32 weeks gestation for a sensitivity precision of 0.15 and indirectly, a specificity precision of 0.034. Through the recruitment of 667 women, we would be able to generate a precise ±2% confidence interval around the estimated GDM prevalence. All analyses were conducted using Stata Statistical software package (StataCorp, College Station, TX).

## Results and discussion

### Results

We exceeded our recruitment target by recruiting 935 eligible pregnant women between July 2013 and August 2015, of whom 886 completed day 1 testing (Fig. 1). Baseline characteristics for all enrolled women are presented in Table 1. 250 (28.9%) enrolled participants did not come back for the day 2 return visit which required fasting, while 616 (71.1%) completed day 1 and day 2 testing. There were no significant differences in demographic characteristics between women who only completed day 1 testing and those who completed both days.

**Table 1:** Characteristics of women who attended non-fasting and fasting visit

	Completed Only Day 1 testing (*N* = 250)	Completed Day 1 and 2 testing (*N* = 616)
Study Location		
MTRH	145	283
UGDH	105	326
Mediheal	0	7
Age (years) (SD)	25.8 (±4.8)	26.1 (±5.1)
Estimated Gestational Age (weeks) (SD)	28.2 (±2.8)	28.3 (±2.6)
Weight (kg) (SD)	61.6 (±10.5)	68.9 (±12.2)
Average SBP (mm/Hg) (SD)	115.8 (±13.5)	112.6 (±18.1)
Average DBP (mm/Hg) (SD)	72.5 (±9.9)	70.8 (±12.9)
SBP ≥ 140 mmHg and/or DBP ≥90 mmHg N(%)	14 (5.6%)	37 (6.0%)
Family history of DM N(%)	38 (15.2%)	94 (15.3%)
Known history of GDM N (%)	0 (0%)	0 (0%)
Average Haemoglobin	N/A	12 (±1.5)
Haemoglobin < 8 N (%)	N/A	14 (2.3%)

*MTRH* Moi Teaching and Referral Hospital, *UGDH* Uasin Gishu District Hospital, *SBP* Systolic Blood Pressure, *DBP* Diastolic Blood Pressure, *DM* Diabetes mellitus, *GDM* Gestational Diabetes Mellitus

Among women who completed day 1 and day 2 testing, the mean age was 26.1 years and mean gestational age was 28.3 weeks. 94 (15.3%) reported a family history of diabetes while 37 (6%) had a systolic and/or diastolic blood pressure above the cut-off for hypertension diagnosis. No patients reported a known history of GDM.

Among women who completed day 1 and day 2 testing, the mean venous fasting glucose was 4 mmol/L (72.0 mg/dL) and mean venous HbA1c was 5.2% (33 mmol/mol). Mean values for the 1 h and 2 h venous glucose were 5.9 mmol/L (106.2 mg/dL) and 5.55 mmol/L (99.9 mg/dL) respectively. Means for the rest of the glucose testing procedures are presented in Table 2.

**Table 2:** GDM Prevalence and blood glucose results

	Completed Only Day 1 testing (*N* = 250)	Completed Day 1 and 2 testing (*N* = 616)
Number (%) diagnosed with GDM based on venous 75 g OGTT IADPSG criteria		18 (2.9%)
Average POC RBG (SD)	4.7 (±1.0)	4.8 (±1.0)
Average POC result after 50 g glucose load (SD)	6.6 (±1.4)	6.9 (±1.4)
Average POC FBG (SD)	N/A	4.2 (±0.6)
Average venous FBG (SD)	N/A	4.0 (±0.5)
Average POC HbA1c (SD)	5.1 (±0.8)	5.2 (±0.5)
Average venous HbA1c (SD)	N/A	5.2 (±0.5)
Average POC 1 h blood glucose post 75 g (SD)	N/A	6.9 (±1.4)
Average venous blood glucose 1 h post 75 g (SD)	N/A	5.9 (±1.4)
Average POC blood glucose 2 h post 75 g (SD)	N/A	6.1 (±1.2)
Average venous blood glucose 2 h post 75 g (SD)	N/A	5.5 (±1.1)

*MTRH* Moi Teaching and Referral Hospital, *UGDH* Uasin Gishu District Hospital, *SBP* Systolic Blood Pressure, *DBP* Diastolic Blood Pressure, *DM* Diabetes mellitus, *GDM* Gestational Diabetes Mellitus, *POC* point of care, *RBG* Random blood glucose, *FBG* Fasting blood glucose; All blood glucose results presented are in mmol/L, hemoglobin presented in g/dl

Eighteen women met IADPSG GDM diagnostic criteria, resulting in an overall GDM prevalence of 2.9% (95% CI, 1.57% - 4.23%) when including only the women who completed day 1 and day 2 testing in our analysis. The majority of GDM patients were diagnosed through a venous FBG whereby 12 of the women met criteria for venous FBG 5.1 mmol/L (91.8 mg/dL) but not 1 h- or 2-h 75 g OGTT cut-off values. 2-h OGTT venous glucose ≥10 mmol/L (180.0 mg/dL) and 1-h OGTT venous glucose ≥8.5 mmol/L (153.0 mg/dL) solely identified an additional 3 and 1 patients respectively. The remaining 2 women were positive on all three IADPSG GDM criteria. These 18 GDM positive women had a median weight of 72.3 kg, median age of 28 years, median gestational age of 28 weeks, 5.5% (*N* = 1) with a family history of diabetes, median systolic blood pressure of 117 mmHg, and median diastolic blood pressure of 70 mmHg. Statistical evaluation of the significance of differences between the GDM positive population and non-GDM population was limited by the small sample of GDM positive women.

The sensitivity, specificity, NPV and PPV of the various screening strategies are shown in Table 3. POC screening strategies showed low sensitivity with the POC 75 g OGTT strategy (‘POC IADPSG’) having the highest sensitivity of 56% and a specificity of 91%. The 1-h post 50 g glucose load POC had a sensitivity of 44% when utilising a cut-off of 7.8 mmol/L (140.4 mg/dL) which increased to 56% on adjusting the cut-off to 7.2 mmol/L (129.6 mg/dL). The specificity of this lower cut-off however dropped from 79% to 64%. Of the non-POC strategies, venous FBS was the most accurate with a sensitivity of 77% and specificity of 100%. When applying the venous HbA1c diagnostic criteria of ≥6.5%, 10 women were identified as GDM positive, however only 30% of these women were positive via the venous 75 g OGTT. The POC HbA1c also demonstrated limited value for identifying women who would be positive on the venous 75 g HbA1c as it was found to have a sensitivity of only 22% despite having a specificity of 99%.

**Table 3:** Performance of various screening strategies

	Sensitivity TP/(TP + FN) 95% CI	Specificity TN/(TN + FP) 95% CI	Negative Predictive Value TN/(TN + FN)	Negative likelihood ratio Point estimate 95% CI	Positive Predictive Value TP/(TP + FP)	Positive likelihood ratio Point estimate 95% CI
Venous 75 g OGTT^a^	1 18/(18 + 0) (0.82–1)	1 598/(598 + 0) (0.99–1)	1 598/(598 + 0)	n/a	1 (18/18 + 0)	n/a
POC RBG ≥ 7 mmol/L (126.0 mg/dL)	0.17 3/(3 + 15) (0.06–0.39)	0.97 581/(581 + 17) (0. 95–98)	0.97 581/(581 + 15)	0.86 (0.70–1.1)	0.15 3/(3 + 17)	5.86 (1.89–18.23)
POC 1 h 50 g ≥ 7.8 mmol/L (140.4 mg/dL)	0.44 8/(8 + 10) (0.25–0.66)	0.79 470/(470 + 128) (0.75–0.82)	0.98 470/(470 + 10)	0.71 (0.47–1.07)	0.06 8/(8 + 128)	2.08 (1.21–3.56)
POC 1 h 50 g ≥ 7.2 mmol/L (129.6 mg/dL)	0.56 10/(10 + 8) (0.34–0.75)	0.64 382/(382 + 216) (0.60–0.67)	0.98 382/(382 + 8)	0.70 (0.41–1.17)	0.04 10/(10 + 216)	1.54 (1.00–2.36)
Venous FBG ≥ 5.1 (91.8 mg/dL)	0.78 14/(14 + 4) (0.55–0.91)	1 598/(598 + 0) (.99–1)	0.99 598/(598 + 4)	n/a	1 14/(14 + 0)	0.22 (0.09–0.53)
POC FBG ≥ 5.1 mmol/L (91.8 mg/dL)	0.33 6/(6 + 12) (0.16–0.56)	0.95 567/(567 + 31) (0.93–0.96)	0.98 567/(567 + 12)	0.70 (0.51–0.98)	0.16 6/(6 + 31)	6.43 (3.08–13.45)
POC 75 g OGTT	0.56 10/(10 + 8) (0.34–0.75)	0.91 542/(542 + 56) (0.88–0.93)	0.99 542/(542 + 8)	0.49 (0.29–0.82)	0.15 10/(10 + 56)	5.93 (3.66–9.61)
POC HbA1c ≥ 6.5%	0.22 4/(4 + 14) (0.09–0.45)	0.99 592/(592 + 6) (0.98–0.99)	0.98 592/(592 + 14)	0.79 (0.61–1.01)	0.4 4/(4 + 6)	22.15 (6.84–71.72)
Venous HbA1c ≥ 6.5%	0.11 2/(2 + 16) (0.03–0.33)	0.99 591/(591 + 7) (0.98–0.99)	0.97 591/(591 + 16)	0.90 (0.76–1.06)	0.22 2/(2 + 7)	9.49 (2.12–42.54)

*TP* true positives, *FN* false negatives, *TN* true negatives, *FP* false positives, *HbAIc* glycated hemoglobin, *OGTT* oral glucose tolerance test, *POC* point of care, *RBG* random blood glucose, *FBG* fasting blood glucose

^a^All results were compared to the venous 75 g oral glucose tolerance test to demonstrate diagnostic accuracy

**Fig. 2:**
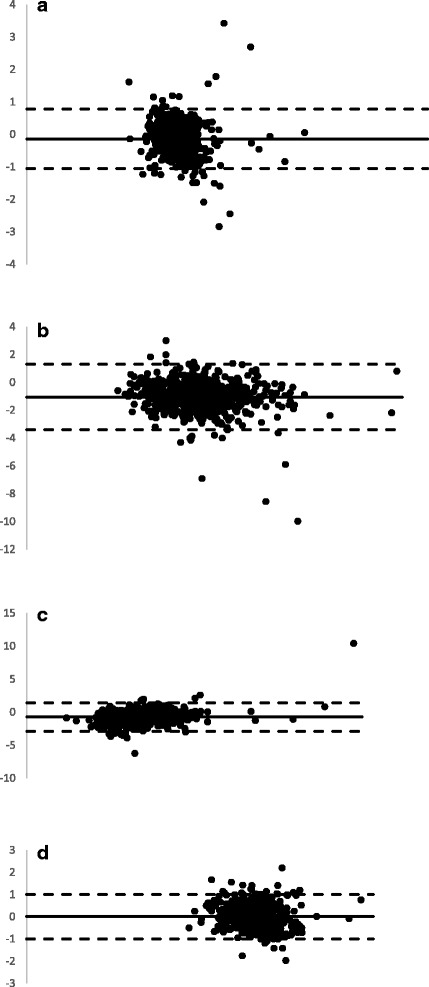
Bland Altman Plots of the Venous and POC Testing Strategies Utilized in this Study. **a** - Fasting Blood Glucose, Venous vs. POC, Correlation coefficient 0.56, *p* < 0.001. **b** - 1 h post 75 g glucose load, Venous vs. POC, correlation coeffiecient = 0.72, *p* < 0.001. **c** - 2 h post 75 g glucose load, Venous vs. POC, correlation coefficient = 0.6910, *p* < 0.001. **d** - HbA1c, Venous vs. POC, correlation coefficient = 0.49, *p* < 0.001

We found a MARD in the test results between POC testing and the corresponding venous testing method of 9.3%,19.4%, 16.0%, and 7.7% for fasting, 1-h, 2-h, and HbA1c testing respectively. The correlation coefficients and *p*-values for the fasting, 1-h, 2-h, and HbA1c values were 0.56 (*p* < 0.001), 0.72 (*p* < 0.001), 0.69 (*p* < 0.001), and 0.49 (*p* < 0.001) respectively. Fasting glucose had the best performance in terms of the MARD despite having the lowest correlation coefficient of the three. This was attributed to the larger impact small variations had on the correlation coefficient at lower glucose values as seen in the Bland Altman plot of the fasting results (Fig. 2a), where the y-axis measurements shows a relatively smaller difference between the two methods of glucose measurement. The unpredictability of the variability is also illustrated in Fig. 2a-d, as the results are scattered above and below zero on all four of the plots illustrating that POC results could be higher or lower than venous results.

Among the 18 women who were positive for GDM based on IADPSG OGTT criteria, 5 (26.3%) were able and willing to provide details on their delivery and return for a follow-up fasting glucose at least six weeks after delivery. None of these women were found to have diabetes upon repeat screening. An additional 6 (31.6%) women were contacted only by phone with the remaining 7 (42.1%) women being completely unreachable. The average birth weight for the 11 women providing data was 3.4 kg with one woman having a macrosomic infant weighing 4.9 kg without any other complications. An additional 2 women underwent Cesarean delivery with one of the babies requiring neonatal intensive care unit admission for an unspecified reason. There was 1 woman who suffered from an intrauterine fetal death which was thought to be unrelated to GDM. Perineal trauma was experienced by 3 additional women without any other clinical sequelae.

### Discussion

We found the prevalence of GDM among pregnant women receiving care in Western Kenya to be 2.9%. We used the IADPSG diagnostic criteria to determine prevalence, a strategy associated with a 2.4 times higher case detection compared to older methods [22]. Our low prevalence may be due to several factors. Firstly, we employed a universal screening strategy among pregnant women regardless of pre-existing risk factors. Secondly, among enrolees, risk factors such as previous GDM, advanced maternal age and elevated blood pressure were low, although, family history of DM was reported in 15.3% of women. Thirdly, our study population represented a mixed population of urban and rural women. GDM risk factors in urban populations include overweight and obese body mass index (BMI), sedentary lifestyle and poor dietary habits. Malnutrition during pregnancy, on the other hand, may be a risk factor for GDM in rural patients [23]. The majority of our enrollees were recruited from two public hospitals which predominatly serve a rural population from farming communities who are less likely to be obese and more likely to be active. We therefore posit that such risk factors were less likely in our study population although complete socio-economic evaluation and determination of BMI are required for firm conclusions to be made.

POC GDM screening strategies performed poorly when trying to approximate the women who would be GDM positive based on the venous 75 g OGTT recommended by IADPSG. The venous 50 g glucose challenge test (GCT) is extensively used as a screening strategy for GDM [24]. We conducted a POC version of the 50 g GCT and found a 56% sensitivity and 64% specificity at a glucose cut-off of ≥7.2 mmol/L (129.6 mg/dL). In contrast, Coustan and colleagues found that the conventional venous 50 g GCT at a glucose cut-off of 6.9 mmol/L (124.2 mg/dL) had a 79% sensitivity and 87% specificity [25]. The POC version of the 50 g GCT may be used as a screening test to identify patients who require furthur testing but its low sensitivity limits its utility. A POC 75 g OGTT strategy had a sensitivity of 56.6% with a specificity of 90.6%. This method may therefore provide reasonable accuracy if a POC strategy is required. However, since this strategy requires for patients to come in fasting, a venous fasting test alone would be a superior approach despite the need for venipuncture as this alone had a 77.7% sensitivity and 100% specificity in our study. For field testing in populations unable to access health facilities regularly however, the POC 75 g OGTT may be used to identify patients who requiring further testing.

POC fasting capillary glucose (FCG) testing using a glucose cut-off of ≥5.1 mmol/L (91.8 mg/dL) in our study had a very low sensitivity of 33.3%. Fadl and colleagues found a slightly higher sensitivity of 47% using an FCG cut-off of ≥5 mmol/L (90.0 mg/dL) among a population at low-risk for GDM [26]. Contralily, Agarwal and colleagues reported a sensitivity of 71.9% and specificity of 76.8% using a FCG cut-off of ≥5.0 mmol/L (90.0 mg/dL) [27]. Their study however, was conducted in a setting with a high prevalence (13.4%) of GDM which may explain the difference in findings.

Additional investigation is required to identify more accurate POC screening strategies for low-risk GDM populations such as ours. Capacity for portable field testing is desirable so as to reach pregnant women unable to access facilities with adequate lab testing resources. Additionally, future evaluation of a feasible screening strategy should also incorporate the study of pregnancy outcomes for all screened women in order to assess the clinical significance of various glucose cut-offs and testing strategies within the context of low and middle-income countries (LMICs). The hyperglycemia and adverse pregnancy outcomes (HAPO) study used this approach to demonstrate that adverse outcomes increase continously from fasting venous glucose values as low as 4.2 mmol/L (75.6 mg/dL) [2]. A similar approach in low-resource populations not included in the HAPO study may be a more accurate method of establishing a feasible and clinically significant screening strategy for GDM. Such an effort would potentially establish population specific cut-off points and contextual testing strategies based directly on pregnancy outcomes within such populations.

Our study has several limitations. Firstly, our low GDM prevalence limited our ability to evaluate different glucose cut-off points for POC testing to establish a clinically useful cut-off with good sensitivity and specificity. We increased the sample size to factor in this unexpectedly low GDM prevalence, we were nevertheless unable to identify the number of cases necessary to meet our prespecified threshold. However, based on the limited concordance found between the various POC strategies tested and the reference IADPSG criteria, the results of our investigation are unlikely to change. Secondly, we found considerable variability between POC test results and corresponding venous test results. This variability increased when patients were tested in non-fasting conditions although we could not find a predictable factor in this variation which we could use to adjust the POC values. Consequently, this may have further limited our ability to draw firm conclusions as far as POC screening strategies were concerned. This study was conducted in a setting that is 2100–2700 m (7000–9000 ft) above sea level, where the high altitude may affect the accuracy of POC glucose testing [28]. Lastly, we were unable to follow up the pregnancy outcomes for all screened mothers, a strategy that may more accurately identify glucose cut-off points with significant clinical outcomes [2]. Because of this limitation and the desire to obtain pregnancy outcomes related to GDM in SSA, we are currently undertaking a larger scale study where we hope to identify Kenyan specific risk thresholds related to glucose screening cutpoints as done within the HAPO study.

## Conclusion

We found a low prevalence of GDM (2.9%) in our setting of Eldoret, Western Kenya. The POC GDM screening strategies we evaluated, although feasible for the universal GDM screening of pregnant women in low resource settings, were found to have a low sensitivity in our study population. Amongst the venous tests evaluated, the majority of GDM positive patients (77.8%) were identified by FBG testing. Further investigations are required to identify accurate POC GDM screening strategies with an emphasis on adverse pregnancy outcomes as an endpoint.

## Abbreviations

ANC: Antenatal clinic
FBG: Fasting blood glucose
FCG: Fasting capillary glucose
FN: False negatives
FP: False positives
GCT: Glucose challenge test
GDM: Gestational diabetes mellitus
HAPO: Hyperglycemia and adverse pregnancy outcomes
HbA1c: Glycated hemoglobin
IADPSG: International association of diabetes and pregnancy study group
LMIC: Low and middle income countries
MARD: Mean absolute relative difference
MTRH: Moi Teaching and Referral Hospital
NPV: Negative predictive value
OGTT: Oral glucose tolerance test
POC: Point of care
PPV: Positive predictive value
RBG: Random blood glucose
SD: Standard deviation
SSA: Sub-Saharan Africa
T2DM: Type 2 diabetes mellitus
TN: True negatives
TP: True positives
UGDH: Uasin Gishu District Hospital
WHO: World Health Organisation
